# Differences in Auditory Distraction between Adults and Children: A Duplex-mechanism Approach

**DOI:** 10.5334/joc.15

**Published:** 2018-02-13

**Authors:** Tanya N. Joseph, Robert W. Hughes, Patrik Sörqvist, John E. Marsh

**Affiliations:** 1 School of Psychology, University of Central Lancashire, Preston, UK; 2 Department of Psychology, Royal Holloway, University of London, Egham, London, UK; 3 Department of Building, Energy and Environmental Engineering, University of Gävle, Gävle, SE

**Keywords:** Attention, Cognitive Control, Development of cognition, Working memory

## Abstract

Differences in the impact of irrelevant sound on recall performance in children (aged 7–9 years old; *N* = 89) compared to adults (aged 18–22 years old; *N* = 89) were examined. Tasks that required serial rehearsal (serial and probed-order recall tasks) were contrasted with one that did not (the missing-item task) in the presence of irrelevant sound that was either steady-state (a repeated speech token), changing-state (two alternating speech tokens) and, for the first time with a child sample, could also contain a deviant token (a male-voice token embedded in a sequence otherwise spoken in a female voice). Participants either completed tasks in which the to-be-remembered list-length was adjusted to individual digit span or was fixed at one item greater than the average span we observed for the age-group. The disruptive effects of irrelevant sound did not vary across the two methods of determining list-length. We found that tasks encouraging serial rehearsal were especially affected by changing-state sequences for both age-groups (i.e., the changing-state effect) and there were no group differences in relation to this effect. In contrast, disruption by a deviant sound—generally assumed to be the result of attentional diversion—was evident among children in all three tasks while adults were less susceptible to this effect. This pattern of results suggests that developmental differences in distraction are due to differences in attentional control rather than serial rehearsal efficiency.

It is long established that serial short-term memory is particularly susceptible to disruption by task-irrelevant sound (Colle & Welsh, 1976; Jones & Macken, 1993; Salamé & Baddeley, 1982). Interest in the present article centres on developmental differences (children compared to adults) in such auditory distraction (e.g., Elliott, 2002) and the extent to which a duplex-mechanism framework (Hughes, 2014; Hughes, Vachon, & Jones, 2007) may help in identifying the basis of these differences. In this framework, there are two distinct mechanisms of auditory distraction: *interference-by-process*, in which serial rehearsal processes deployed to perform the recall task are disrupted by the obligatory seriation of a sound sequence (e.g., Jones & Macken, 1993), and *attentional diversion* in which the sound draws attention away from the task (Hughes, 2014; Hughes, Vachon, & Jones, 2005, 2007). By manipulating not only the nature of the irrelevant sound sequence but also the likelihood that serial rehearsal will be used to perform the focal recall task, we sought to examine whether children are more susceptible to auditory distraction than adults due to under-developed rehearsal ability or to under-developed attentional control.

The interference-by-process mechanism within the duplex-mechanism account of auditory distraction is witnessed most commonly in the form of the *changing-state effect* in the context of a visually-presented verbal serial recall task. Here, around six to eight verbal items (e.g., letters or digits) are presented one by one on a screen (at about one item per second) and which, following the last item, must be recalled in strict serial order. When an irrelevant sound (e.g., speech) sequence is presented in the background, serial recall is disrupted markedly but only if the sound contains acoustic variation from one element to the next (“*A-B-A-B*…”); with a steady-state sound (“*A-A-A-A*…”) little or no disruption is produced (e.g., Campbell, Beaman, & Berry, 2002; Jones & Macken, 1993; Jones, Madden, & Miles, 1992; Schlittmeier, Weisz, & Bertrand, 2011). It is argued that the changes in the sound yield order cues that are processed obligatorily and interfere with the similar process of seriating the to-be-remembered items in the form of serial rehearsal (e.g., Jones & Macken, 1993). In comparison, a steady-state sound causes minimal or no disruption because there are no order cues being generated. Importantly, therefore, this form of distraction is a joint product of the involuntary processing of the sound and a particular, deliberate, process (serial rehearsal) being used to perform the focal task (Jones & Tremblay, 2000).

The attentional diversion component of the duplex-mechanism account has been studied mainly through the disruptive impact on serial recall of an unexpected deviant sound embedded within an irrelevant sound sequence. For example, if one token in a speech sequence is presented in a different voice from the remainder (e.g., *A-B-A-B-A*…; with the token in bold indicating a male-spoken ‘deviant’ token embedded in an otherwise female-spoken sequence), serial recall is disrupted over and above any changing-state effect (Hughes et al., 2005, 2007; Hughes, Hurlstone, Marsh, Vachon, & Jones, 2013). It is argued that this *deviation effect* reflects the diversion of attention away from the focal task due to the fact that it violates expectancies based on the prevailing pattern of auditory input (e.g., Hughes et al., 2005; Vachon, Hughes, & Jones, 2012).

There is ample evidence for the dichotomy between the interference-by-process and attentional diversion mechanisms embodied in the duplex-mechanisms account, based primarily on empirical dissociations between the changing-state effect and the deviation effect. First, the effects differ with regards to task sensitivity. By definition, the interference-by-process explanation of the changing-state effect supposes that the focal task must involve a seriation process in order for it to be vulnerable to the similar process of seriating the changing-state sound. And indeed, whereas serial-order based tasks exhibit a marked changing-state effect, it is typically attenuated if not absent in non-seriation based memory tasks (Elliott et al., 2016; Hughes et al., 2007; Jones & Macken, 1993; Marsh et al., in press). For example, when participants are asked to identify the item missing from a list drawn from a well-known fixed set (e.g., *2* is missing from the list *59384716*), the changing-state effect is absent (so long as participants do not happen to adopt a serial rehearsal strategy to perform the task; Hughes & Marsh, 2018). In contrast, the deviation effect is observed not only in serial recall but also in non-seriation tasks, including the missing-item task (e.g., Hughes et al., 2007; Vachon, Labonté, & Marsh, 2017; Parmentier, 2008). Second, the effects also differ in their amenability to cognitive control: Whereas the deviation effect can be attenuated by top-down cognitive control, the changing-state effect appears to bypass such control. For instance, when greater task-engagement is promoted by making the encoding of the to-be-remembered items more difficult or when a forewarning about the nature of the distraction is provided, the deviation effect but not the changing-state effect is eliminated (Hughes et al., 2013; but see Röer, Bell, & Buchner, 2015). This pattern is in line with the notion that it is the very act of performing the (serial-order based) task that renders it susceptible to the changing-state effect while the deviation effect reflects a momentary disengagement from a task. Third, individuals with low working memory capacity (considered a measure of attentional control; e.g., Engle & Kane, 2004) are particularly susceptible to the deviation effect but not the changing-state effect (Hughes et al., 2013; Sörqvist, 2010; Sörqvist, Marsh, & Nöstl, 2013; but for a recent failure to replicate this result, see Körner, Röer, Buchner, & Bell, 2017).

In the present study, we use the duplex-mechanism account as a framework for trying to understand the well-documented finding that children are more susceptible to auditory distraction than adults (e.g., Elliott, 2002; Elliott, Bhagat, & Lynn, 2007; Elliott et al., 2016; Klatte, Lachmann, Schlittmeier, & Hellbrück, 2010). In particular, we exploit the different ways in which a changing-state sound sequence and a deviant sound are thought to disrupt short-term memory to examine the possible roles of, respectively, serial rehearsal ability and attentional control in children’s increased susceptibility. Children’s attentional control begins to emerge early in life (towards the end of the first year; Deoni, Mercure, Blasi, Gasston, Thomson, & Johnson, 2011) but still by around seven years of age, the ability to exert top-down control by inhibiting irrelevant information remains markedly less efficient than in adults (Davidson, Amso, Anderson, & Diamond, 2006; Hwang, Velanova, & Luna, 2010). The maintaining of information whilst concurrently inhibiting irrelevant information is thought to be a function of working memory (Engle & Kane, 2004; Kane, Conway, Hambrick, & Engle, 2008). Indeed, it has been suggested that individual differences in working memory capacity (WMC) are in fact individual differences in attentional control, with individuals with high WMC better able to control the contents of working memory (Conway et al., 2001; Sörqvist, 2010). Therefore, children’s lower working memory capacity (Cowan, Elliott, Saults, Morey, Mattox, Hismjatullina, & Conway, 2005) may be responsible for their greater susceptibility to auditory distraction. If so, based on the duplex-mechanism account, we would expect children to be particularly susceptible to distraction that specifically reflects attentional diversion (e.g., the deviation effect).

However, it is well recognized that children’s rehearsal abilities also develop through childhood, changing from individual-item labelling in early childhood to a cumulative style of rehearsal in later childhood and into adulthood (Jarrold & Hall, 2013; Lehmann & Hasselhorn, 2012; Tam, Jarrold, Baddeley, & Sabatos-DeVito, 2010; Tucker-Drob, 2009). Thus, to the extent that children deploy serial rehearsal, its inchoate state may also underpin an increased susceptibility to the changing-state effect in the context of serial-order based recall tasks.

There already exists some evidence suggesting that children’s increased susceptibility is due to a greater vulnerability to attentional diversion rather than interference-by-process. For example, Elliott et al. (2016) found that children (aged around 7) were no more susceptible to the changing-state effect than adults but that they were more susceptible to irrelevant sound generally. That is, children were susceptible to steady- as well as changing-state sound and this occurred regardless of whether the focal task encouraged a serial rehearsal strategy. They concluded that these general effects of sound were most readily explained in terms of a greater likelihood of attentional diversion in children (for similar conclusions, see Klatte et al., 2010). However, this inference is somewhat indirect: the general effects of sound in children were attributed to attentional diversion on the grounds that they were shown not to be changing-state effects.

In the present study, we take a more direct approach to this issue by examining for the first time whether children (aged 7–9 years) show a particular sensitivity to the deviation effect, an effect that can be more unambiguously attributed to attentional diversion (e.g., Hughes et al., 2007; Korner et al., 2017). At the same time, we again examine whether or not children and adults are equally susceptible to the changing-state effect. Our decision to test 7–9 year olds in our study was based on evidence that at this age children’s ability for serial rehearsal is immature (Lehmann & Hasselhorn, 2007, 2012) and so the possibility that they could be more sensitive to auditory distraction due to a greater susceptibility to interference-by-process could be examined. Testing children aged 10 or older may have meant missing a potentially key difference between children and adults as there is evidence that by this age children are adept at using serial rehearsal (Lehmann & Hasselhorn, 2007, 2012). Conversely, there is evidence that children do not use serial rehearsal before age 7 (Flavell, Beach, & Chinsky, 1966; Henry, 1991; but see Jarrold & Hall, 2013) and so testing children younger than this may not have been suitable for addressing the increased-interference-by-process hypothesis.

Specifically, then, we contrasted children’s short-term memory performance under conditions of steady- and changing-state sound sequences and, within each of these sequence-types, a deviant-voice token was presented relatively infrequently across the block of trials. At the same time, we manipulated the likelihood of the involvement of serial rehearsal in the focal task by contrasting the effect of these sound conditions not only on serial recall but also the missing-item task which, as noted, is widely thought not to be serial-rehearsal based. Furthermore, we included a second order-based recall task—probed-order recall—that arguably provides a better match to the response-demands of the missing-item task than serial recall (cf. Beaman & Jones, 1997; Elliott et al., 2016; Hughes, Marsh, & Jones, 2011). We predicted that the deviation effect would not vary as a function of task and that children would show a greater sensitivity to this effect than adults. In contrast, the changing-state effect—which should only be apparent in the two serial-order based tasks (serial recall and probed-order recall)—should not differ as a function of age-group.

Finally, within each task, we used two approaches to trying to equate task difficulty across the two age-groups: The length of the to-be-remembered list was determined either according to each participant’s digit span or according to the average digit span for each age-group. Attempting to equate task-difficulty between the groups was important as there is evidence that one of the forms of auditory distraction (attentional diversion, as manifested in the deviation effect) is attenuated under greater focal-task difficulty (e.g., Hughes et al., 2013); thus, any difference in the deviation effect that might be found as a function of age-group could be confounded with greater task-difficulty unless the latter factor is controlled.

## Method

### Participants

Eighty-nine children who were pupils at a number of schools in Lancashire, UK, and aged 7–9 years old (47 females; *M* = 8 years, 2 months) and 89 undergraduate students at the University of Central Lancashire aged 18–22 (69 females; *M* = 20 years) participated in this study. Forty children and 39 adults completed a span-adjusted version of the three memory tasks (see below) while 49 children and 50 adults completed a fixed list-length version of the tasks (for more detail, see below). Adults received a small honorarium for their participation while children were given stickers.

### Apparatus and Materials

All tasks were run on a desktop computer (Belinea BB10002) or laptop (Lenovo ThinkPad E560) using E-Prime 2.0 software (Psychology Software Tools). The screen size (and resolution) was 17" (1920 × 1080 pixels) and 15.6" (1280 × 1024), respectively, for the desktop and laptop computers. Sennheiser HD-202 headphones were used to present the auditory sequences.

**To-Be-Remembered Lists.** The to-be-remembered items for all tasks were drawn from the set 0–9. The digit sequence for each trial was randomly generated using MATLAB, ensuring that digits were sampled without replacement and with the constraint that no sequence had three or more consecutive digits in ascending or descending order (e.g., …*4, 3, 2*…). Each to-be-remembered item appeared in black 72 point *Arial* font on a white background for 1000 ms with no inter-stimulus interval.

**Irrelevant Sound Sequences.** The sound sequences were recorded using a broadcast quality Dictaphone in a sound attenuated chamber and then edited using *Sony Sound Forge Pro 11* software (Sony Creative Software). Four spoken items were recorded with a 16-bit resolution and a sampling rate of 44.1 KHz. These were the letters ‘A’ and ‘B’ in both a female and a male voice. These were then digitally edited to 250 ms and used to construct four types of sound sequence: changing-state (*ABAB…or BABA*…all in a female voice), steady-state (*AAAA*… or *BBBB*…all in a female voice), a changing-state sequence with one of the female-spoken items replaced with a ‘deviant’ male-spoken item (e.g., *ABA****B****A*…; male-spoken item shown in bold) and a deviant male-spoken item within a steady-state sequence (e.g., *AAA****A****A*…). In each sequence, there was a 500 ms inter-stimulus interval and there were two sound tokens per to-be-remembered item. The onset of the first sound coincided with that of the first to-be-remembered item. For with-deviant trials, the deviant occurred either exactly or as close as possible to 5/8ths of the way through the to-be-remembered list (cf. Hughes et al., 2013) regardless of list-length. For example, with a list-length of 8, the deviant was the 10^th^ out of the 16 sounds in the sequence, with a list-length of 5, the deviant was the 6^th^ out of 10 sounds, and with a list-length of 4 the deviant was the 5^th^ of 8 sounds. A quiet condition was also included, making a total of five auditory conditions. Sounds were played at approximately 55 dB(A) as measured with a sound level meter and an earphone coupler.

#### Recall Tasks

***Digit span test*.** The digit span test was used to assess a participant’s verbal short-term memory capacity. Participants were shown digits on screen and were required to recall them in the order they were presented. There were three trials of each set-size ranging from three to nine items (taken without replacement from the set 0–9). An individual’s digit span was determined as the last set size at which all items were correctly recalled in order on at least two of the three trials (testing stopped for a given participant at this point also).

***Serial recall*.** Participants were shown a list of digits drawn from 0–8 on screen at the rate of one digit every second. Following the last item, the possible response candidates for that list were presented in canonical order and participants were required to click on the digits in the order they had been presented. There was no time-limit on recall and the cue for the next trial (‘Begin’) appeared when all the response boxes were filled. Participants clicked on ‘Begin’ to commence to the next trial.

***Probed-order recall task*.** In this task, digit presentation was identical to the serial recall task but at the end of the list the question ‘Which number followed *x* in the list?’ was displayed for 1000 ms, where *x* could be any of the just-presented items (except the last one). This was followed by the canonical digit array. Once the response was made, the word ‘Begin’ appeared for participants to click to initiate the next trial.

***Missing-item task*.** In this task, participants were instructed that a fixed list of digits would be shown on screen (e.g., digits from 0–5) and that one digit would be missing from this list on each trial. Their task was to identify the missing digit. At the end of each list, the question ‘Which number was missing from the list?’ was displayed on the screen for 1000 ms followed by the digits from the relevant set presented in a canonically ordered array. Each digit was missing roughly an equal number of times. After the response was made, the word ‘Begin’ appeared for participants to initiate the next trial.

### Design

The experiment had a mixed-factors design with Task-type (serial recall, probed-order recall, missing-item) and Sound condition [quiet, steady-state (SS), changing-state (CS), steady-state+deviant (SS+d), changing-state+deviant (CS+d)] as within-participant factors and age-group (children, adults) as the between-participants factor. Each recall task had two blocks of trials. For adults, one of these blocks contained 8 quiet trials, 16 CS trials, and 8 CS+d trials arranged in a pseudo-random order with the constraint that no condition was represented more than twice across immediately successive trials. A randomly determined half the CS sequences took the form ‘ABABA…’ and the other half the form ‘BABAB…’ and so the deviant-voice item was equally likely to be an ‘A’ or a ‘B’. The other block contained 8 quiet trials, 16 SS trials, and 8 SS+d trials, arranged with the same constraints as the other block. A randomly determined half the SS sequences took the form ‘AAAAA…’ and the other half took the form ‘BBBBB…’ so again the deviant-voice item was equally likely to be an ‘A’ or a ‘B’. The children received the same trial-block structure but the numbers of trials per condition in a block in this case were, respectively, 6, 12, and 6 for the quiet, CS (or SS), and CS+d (or SS+d) conditions. Regardless of age-group, the order of the two blocks was counterbalanced across participants.

### Procedure

The tasks were administered on desktop computers for adult participants. They were tested in a quiet lab in groups of up to four people and the testing session lasted roughly 60–70 min. Children were tested in a quiet classroom arranged to accommodate six children at a time and laptops were used for these sessions. The children’s testing sessions were conducted over four weeks such that each task was administered over the course of approximately a week. Participants were seated at a viewing distance of approximately 60 cm from the display monitor and headphones were worn for the duration of each memory task. They were instructed to ignore the sounds played through the headphones and reassured that they would not be tested on it at any point during the study. Half way through each recall task, participants were informed by an on-screen message that they could take a short break and would need to press the Space Bar to continue to the next part.

In the span-adjusted version of each of the three tasks, the to-be-remembered list-length was based on each participant’s digit span. The to-be-remembered list for participants with a span length of 3 comprised three digits drawn from the four-item set 0–3; for span length 4, four digits were drawn from 0–4, and so on. The average span was 4 items for children and 7 items for adults. Thus, the list-length for the fixed-length version of the tasks was set at one item greater than the average span for each age-group (i.e., 5 and 8 items) and the digits were drawn from the sets 0–5 and 0–8, respectively.

## Results

**Figure 1:**
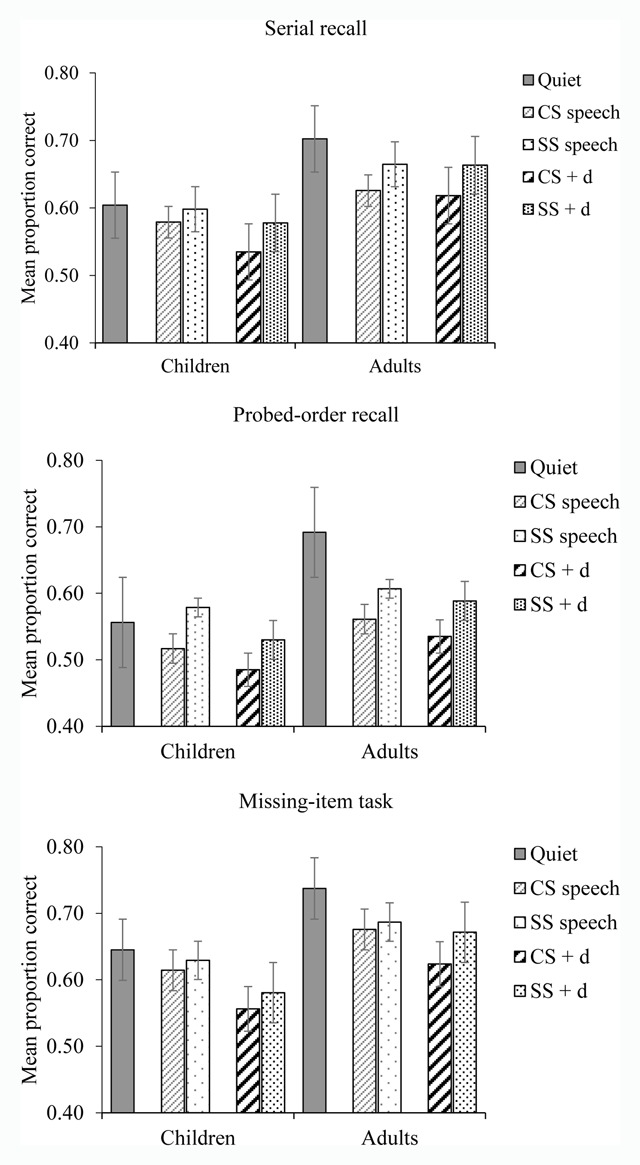
Recall performance in each task by auditory condition and age-group collapsed across span-adjusted and fixed length methods. Error bars represent the standard error of the mean.

For the serial recall task, the data were scored according to the standard strict serial recall scoring procedure: an item was only scored as correct if it corresponded to the absolute serial position in which it was presented. The mean proportion of correct responses averaged across serial position was then used for the analyses. The data for both the probed-order recall and the missing-item task was the proportion of trials on which the correct item was recalled/identified.^1^ Figure 1 shows mean recall performance of children and adults (collapsed across the two methods of determining list-length as this did not interact with the key factors of interest; see below) in each of the five auditory conditions in each task.

We first conducted a 5(Sound condition: Quiet, CS, SS, CS+d, and SS+d) × 3 (Task: serial recall, probed-order recall, missing-item task) × 2 (List-length method: span-adjusted, fixed-length) × 2 (Age-group: children, adults) mixed ANOVA (the full set of results from this analysis is provided in Table 1). Greenhouse-Geisser corrections did not change any of the results. There was a significant main effect of Sound condition, *F*(4, 696) = 37.95, *MSE* = .019, *p* < .001, \eta _p^2 = \,\,.18, reflecting the disruption of performance overall in the presence of sound (means: Q = 67, SS = .64, CS = .61, SS+d = .62, CS+d = .57). Importantly, there was also a significant interaction between Sound condition and Age-group, *F*(1, 174) = 3.87, *MSE* = .02, *p* = .004, \eta _p^2 = \,\,\,.02. Children’s performance was poorer than that of adults in the Quiet (*Ms*: .62 vs. .72), CS+d (.54 vs. .60), and SS+d conditions (.58 vs. .65) but not in any other conditions. There was also a significant main effect of Task, *F*(2, 348) = 23.82, *MSE* = .064, *p* < .001, \eta _p^2\,\, = \,\,.12, wherein performance in the missing-item task (*M* = .66) was significantly better than in the probed-order recall (*M* = .58) and serial recall tasks (*M* = .63); the difference between the latter two tasks was also significant (all *p*s < .05). The main effect of Age-group was also reliable, whereby children’s overall level of recall performance (*M* = .59) was significantly lower than that of the adults (*M* = .65) but this main effect was qualified by its interaction with List-length method, *F*(1, 174) = 6.43, *MSE* = .41, *p* = .012, \eta _p^2 = \,\,.04: The performance of children was only poorer than that of adults with the fixed list-length method and not the span-adjusted method (Fixed method: Children’s *M* = .44 vs. Adults’ *M* = .57; Span-adjusted method: Both groups’ *M* = .74). Thus, the span-adjusted method turned out to be the more effective one for equating overall task-difficulty between the groups. The main effect of List-length method was also reliable, *F*(1, 174) = 88.89, *MSE* = .41, *p* < .001, \eta _p^2\,\, = \,\,.34 (span-adjusted *M* = .74; fixed method *M* = .50) as was the List-length by Task interaction, *F*(2, 348) = 6.78, *MSE* = .064, *p* = .001, \eta _p^2 = \,\,.038, whereby, regardless of age-group, while performance in all tasks benefitted from the span-adjusted method, this was especially the case in the serial recall and the missing-item tasks (Serial recall: Span-adjusted *M* = .76 vs. Fixed *M* = .50; Missing-item: Span-adjusted *M* = .79 vs. Fixed *M* = .52; Probed-order: Span-adjusted *M* = .48 vs. Fixed *M* = .67). It is not clear why this was the case but the most important aspect of the results as far as List-length method is concerned is that it did not enter into any interactions involving Sound condition. That is, even though task-difficulty was greater for the children than adults with the fixed list-length method, this did not modulate the auditory distraction effects or their interaction with age-group.

**Table 1:** Full set of results from the Auditory condition × Task × Age group × List-length ANOVA.

Factor(s)	df	*MSE*	*F*	*p*	\eta _P^2

Auditory condition	4,696	.72	37.95	<.001	.18
Task	2,348	1.53	23.82	<.001	.12
Age group	1,174	2.92	7.09	<.01	.04
List-length	1,174	36.67	88.89	<.001	.34
Auditory condition × Task	8,1392	.03	1.39	.20	.01
Auditory condition × Age group	4,696	.07	3.87	.004	.02
Auditory condition × List-length	4,696	.04	2.11	.078	.01
Task × Age group	2,348	.01	.16	.848	.001
Task × List-length	2,348	.44	6.78	<.005	.04
Age group × List-length	1,174	2.65	6.43	.012	.04
Auditory condition × Task × Age group	8,1392	.01	.77	.628	.004
Auditory condition × Age group × List-length	4,696	.01	.66	.617	.004
Task × Age group × List-length	2,348	.02	.28	.757	.002
Task × Auditory condition × List-length	8,1392	.03	1.48	.160	.01
Task × Auditory condition × List-length × Age group	8,1392	.02	.83	.578	.01

Appendix: Tables showing the full set of results for both ANOVAs.

We then conducted an ANOVA that focused on the key predictions of interest; those involving the changing-state effect and the deviation effect and whether these effects varied as a function of task and age-group. This meant omitting the additional quiet control condition so that the main four sound conditions of interest (SS, CS, SS+d, CS+d) could be grouped according to the manipulation of deviance and state [i.e., 2(Deviation) × 2(State)]. Given that List-length method did not interact with Sound condition in the previous analysis, the data were collapsed across the levels of this factor. Note that we do not mention again here results that were already reported from the initial ANOVA above (e.g., main effect of Task; but again the full set of results can be seen in Table 2). A 3(Task) × 2(State: SS, CS) × 2(Deviation: present, absent) × 2(Age-group: children, adults) mixed ANOVA showed a main effect of State, *F*(1, 176) = 29.52, *MSE* = .025, *p* < .001, \eta _p^2 = \,\,.14, with recall being poorer with changing-state (*M* = .58) compared to steady-state sound (*M* = .62), and a main effect of Deviation, *F*(1, 176) = 30.9, *MSE* = .017, *p* < .001, \eta _p^2 = \,\,.15, with recall also being poorer generally in the presence (*M* = .58) compared to the abence (*M* = .61) of a deviant (i.e., the deviation effect). Contrary to expectations, there was no significant interaction between State and Task, *F*(2, 352) = 1.39, *MSE* = .023, *p* = .25, \eta _p^2 = \,\,.01, but this appears to have resulted from a non-significant trend for a (spurious) changing-state effect in the missing-item task that arose due to a tendency in this experiment for the deviation effect to be larger in a changing-state context (i.e., CS+d) than in a steady-state context (i.e., SS+d). Supporting this interpretation, if the with-deviant conditions are left out, the changing-state effect is significant only in the serial recall task, *p* < .005, and the probed-order recall task, *p* < .001, and not in the missing-item task, *p* = .38. As predicted, there was no interaction between Deviation and Task: In contrast to the changing-state effect, the deviation effect was significant in all three tasks (all *p*s < .02). Of particular interest for present purposes is that there was no interaction between Age-group and State, *F* < 1, while there was, as predicted, an interaction between Age-group and Deviation, such that children exhibited greater vulnerability to the deviation effect than adults, *F*(1, 176) = 3.83, *MSE* = .017, *p* < .03 (one-tailed), \eta _p^2 = \,\,\,.02: Children no-deviant *M* = .54 vs. with-deviant *M* = .59; Adults no-deviant *M* = .64 vs. with-deviant *M* = .62. No other interactions were significant.

**Table 2:** Full set of results from the State × Deviation × Task × Age group ANOVA.

Factor(s)	df	*MSE*	*F*	*p*	\eta _P^2

State	1,176	.75	29.52	<.001	.14
Deviation	1,176	.51	30.90	<.001	.15
Task	2,352	1.20	20.21	<.001	.10
Age group	1,176	2.03	3.92	<.05	.022
State × Deviation	1,176	.02	.92	.339	.01
State × Task	2,352	.03	1.39	.249	.01
State × Age group	1,176	.004	.16	.686	.001
Deviation × Task	2,352	.03	1.47	.230	.01
Deviation × Age group	1,176	.06	3.83	.052	.02
Task × Age group	2,352	.04	.61	.543	.003
State × Deviation × Task	2,352	.01	.52	.597	.003
State × Deviation × Age group	1,176	.004	.24	.627	.001
State × Task × Age group	2,352	.003	.13	.879	.001
Deviation × Task × Age group	2,352	.001	.06	.941	<.001
State × Deviation × Task × Age group	2,352	.01	.41	.661	.002

## Discussion

The present results support the notion that children are particularly susceptible to auditory distraction due to poorer attentional control as opposed to being underpinned by a greater vulnerability to interference-by-process due to under-developed rehearsal ability (cf. Elliott et al., 2016; Klatte et al., 2010; see also Meinhardt-Injac et al., 2015; Wetzel, 2015). First, we replicated the finding that the changing-state effect—the most common signature of interference-by-process—is only observed in the context of order-based tasks (serial recall and probed-order recall but not the missing-item task; Elliott et al., 2016; Jones & Macken, 1993; Hughes et al., 2007) and, second, we confirmed that children are no more susceptible to the changing-state effect than adults (Elliott et al., 2016). Third, the results supported the view that the deviation effect is not task-sensitiveor at least is not restricted to serial-order based tasks—and, fourth, we showed that children were indeed more susceptible to this effect than adults. While determining the list-length for the two age-groups according to their respective average spans disadvantaged the children generally, overall performance was equivalent when list-length was determined according to each participant’s span. Most importantly, list-length method did not interact with the auditory distraction effects or their interaction with age-group and so we can be fairly confident that our results were not distorted by a difference in task-difficulty across age-groups.

While the present pattern of findings supports a duplex-mechanism approach to developmental differences in auditory distraction, it should be acknowledged that some aspects of the present data did not conform entirely to expectations. For instance, we did not replicate Elliott et al.’s (2016) finding that children show greater vulnerability than adults to steady-state sound (compared to quiet). Elliott et al. (2016) interpreted this effect as suggesting that sound generally—regardless of whether or not it changes or contains deviants—could cause attentional diversion in children. One possibility that may merit further investigation is whether the general effect of sound in children depends on the overall auditory context; it seems plausible that the general effect of sound is reduced when the overall experimental session or block contains trials with particularly attention-diverting deviant sounds, as was the case in the present experiment but not in Elliott et al. (2016). In general, further work needs to be done to understand possible block- or session-wide contextual effects on auditory distraction, particularly given the established sensitivity of attentional diversion to cross-trial effects (Vachon et al., 2012). Alternatively, the discrepancy between the present results and Elliott et al. (2016) in relation to the general effect of sound may at least in part be related to the fact that the children were somewhat younger—and hence possibly had poorer attentional control—in the relevant experiment (Experiment 2) of their study (*M* = 7.6 years) compared to ours (*M* = 8.2 years).

A second aspect of the results that deserves discussion is that the deviation effect, while significant overall, was rather small and sporadic in the adult group in the present experiment. While there was no interaction between age-group, deviation and task, it is evident from Figure 1 that adults failed to show a deviation effect in the serial recall task and indeed further statistical analysis revealed that the deviation effect in adults only reached significance in the missing-item task (*p* < .03). One possibility is that the deviation effect was relatively weak in the present experiment because it was particularly amenable to top-down factors that attenuate its potency (cf. Hughes et al., 2013). Specifically, across the experiment, for any given participant, the deviant event could only be either a male-spoken “A” or a male-spoken “B”. This contrasts with the more typical design in which the deviation is a change of voice but the item that is subject to that change is one out of a relatively large set (e.g., Hughes et al., 2007, 2013). It is plausible, with the more constrained deviation as used here, a session/block-wide expectation for that deviant is more readily generated such that the degree to which it violates expectations and diverts attention is reduced (cf. Vachon et al., 2012). On this interpretation, it might be suggested that the child group remained susceptible to even the relatively constrained deviation used here due to a less developed capacity to incorporate the nature of the deviation into a block/session-wide model of the auditory context.

The findings from the present study also indicate that those factors responsible for the developmental increase in span or short-term memory capacity may not be the same as those determining the level of disruption through interference-by-process (Elliott & Cowan, 2005). As children get older, their language proficiency and vocabulary improves which in turn improves their verbal short-term memory performance (Cherney, 2003). Alongside language development is the emergence of rehearsal around the age of seven (e.g., Bebko, 1984; Bjorklund, Coyle, & Gaultney, 1992; Flavell et al., 1966). Taken together, these factors result in greater short-term memory ability, but, as this study and that by Elliott et al. (2016) have shown, they do not directly determine the magnitude of the changing-state effect. This conclusion harmonises with the finding that neither complex span measures (such as operation span)—thought to be a measure of attentional control in addition to short-term memory (Engle & Kane, 2004)—not simple span measures are closely related to the degree of distraction produced by changing-state sounds (Ellermeier & Zimmer, 1997; Sörqvist et al., 2013). In summary, the present results suggest, in keeping with prior findings (e.g., Elliott et al., 2016; Klatte et al., 2010), that children’s greater susceptibility to auditory distraction is due to a greater propensity for attentional diversion in children rather than differences in rehearsal ability. Children have a greater susceptibility to distraction not because of under-developed rehearsal skill giving rise to stronger interference-by-process during serial-order tasks but because poorer levels of attentional control leave them more vulnerable to instances of attentional diversion, and this regardless of the nature of the task in hand. The present data also suggest that more research is needed to examine the role of the overall auditory context and structure on the degree to which a given sound event is endowed with attention-diverting power and how this in turn relates to developmental differences and hence attentional control capacity.

## Data Accessibility Statement

Data are accessible from DOI: https://doi.org/10.17030/uclan.data.00000150

## Footnotes

The data from this study are available at DOI: https://doi.org/10.17030/uclan.data.00000150
